# Characterizing Patients with Unilateral Vestibular Hypofunction Using Kinematic Variability and Local Dynamic Stability during Treadmill Walking

**DOI:** 10.1155/2017/4820428

**Published:** 2017-07-13

**Authors:** Peng Liu, Qiuhong Huang, Yongkang Ou, Ling Chen, Rong Song, Yiqing Zheng

**Affiliations:** ^1^Guangdong Provincial Engineering and Technology Center of Advanced and Portable Medical Devices, School of Engineering, Sun Yat-sen University, No. 132, East Waihuan Rd, Higher Education Mega Center, Guangzhou 510006, China; ^2^Department of Otolaryngology-HNS, Sun Yat-sen Memorial Hospital, Sun Yat-sen University, 107 Yanjiang West Rd, Guangzhou 510120, China

## Abstract

Here, we aimed to compare the unstable gait caused by unilateral vestibular hypofunction (UVH) with the normal gait. Twelve patients with UVH and twelve age-matched control subjects were enrolled in the study. Thirty-four markers were attached to anatomical positions of each participant, and a three-dimensional (3D) motion analysis system was used to capture marker coordinates as the participants walked on a treadmill. The mean standard deviation of the rotation angles was used to represent gait variability. To explore gait stability, local dynamic stability was calculated from the trunk trajectory. The UVH group had wider step width and greater variability of roll rotation at the hip than the control group (*P* < 0.05). Also, the UVH group had lower local dynamic stability in the medial-lateral (ML) direction than the control group (*P* < 0.05). By linear regression analysis, we identified a linear relationship between the short-term Lyapunov exponent and vestibular functional asymmetry. The result implies that UVH-induced asymmetry can increase posture variability and gait instability. This study demonstrates the potential for using kinematic parameters to quantitatively evaluate the severity of vestibular functional asymmetry. Further studies will be needed to explore the clinical effectiveness of such approaches.

## 1. Introduction

Gait stability requires a complex set of sensorimotor controls [1, 2], including sensory inputs, integration of sensory inputs, and motor outputs [3, 4]. Vestibular sensory inputs play an important role in motion acceleration and body orientation [5, 6]. Yamamoto et al. found that participants walked towards the stimulated side after ice water irrigation to the unilateral external auditory canal and noted that the regulation of dynamic head and trunk movement in the medial-lateral (ML) direction was via the horizontal semicircular canals [7]. Besides that, galvanic vestibular stimulation (GVS) has also been used to explore the contribution of the vestibular system during locomotion. Trunk variability and trajectory deviation increased with the onset of GVS [8, 9]. Therefore, we can infer that unilateral vestibular hypofunction (UVH) might affect motor output through the vestibulospinal reflex (VSR) pathway and cause gait disorders.

Gait analysis provides useful information about the physiological status of patients with gait disorders and has been widely applied in the evaluation of osteoarthritis and nervous diseases such as Parkinson's disease [10–12]. Mills et al. explored the relationship between joint angle asymmetries and the progression of knee osteoarthritis [10]. In Parkinson's disease, gait freezing was observed in patients during turning walking [13]. Kinematic variability has been widely used to reflect rhythmicity [10, 14–16]. Gait analysis of patients with small vestibular schwannomas manifested that phase-related variability was associated with the severity of vestibular impairment [17]. To achieve gait stability, body segments should be coordinated with each other to keep the body moving forward smoothly [18].

The short-term Lyapunov exponent, as a nonlinear-time analysis indicator, has been used to quantify the local dynamic stability of a gait pattern [19–22]. Local dynamic stability of walking represents the sensitivity of gait to infinitesimally small perturbations [23, 24] and has been applied to represent fall risk in older people and patients with diabetic neuropathy [19, 21]. Higher short-term Lyapunov exponent indicates greater exponential divergence of gait trajectories, resulting in higher fall risk [25].

However, few nonlinear measurements have been applied to quantify the gait of patients with UVH, and the relationship between the vestibular functional asymmetry and gait disorders remains unclear. Here, we aimed to investigate the gait pattern of patients with UVH by analyses of kinematics and local dynamic stability. Linear regression analysis was used to investigate the relationship between vestibular functional asymmetry and gait disorders.

## 2. Materials and Methods

### 2.1. Subjects

Twelve patients (4 females and 8 males, age: 58.0 ± 10.9 years) with UVH were recruited in the UVH group as well as twelve healthy volunteers (3 females and 9 males, age: 58.8 ± 12.9 years) in the control group, matched according to age. The leg length of the UVH group (0.92 ± 0.04 m) was matched with that of the control group (0.91 ± 0.07 m) to eliminate the impact of body size on gait parameters [26]. The experimental protocols were approved by the Ethics Committee of the Sun Yat-sen Memorial Hospital and all subjects provided with informed consent. All the participants underwent vestibular function tests at the Sun Yat-sen Memorial Hospital, and twelve patients exhibited pure unilateral vestibular deficit. Unilateral vestibular dysfunction was determined by the caloric test, in which the canal paresis (CP) value reflects the degree of asymmetry of bilateral vestibular function. CP value greater than 20% was considered indicative of unilateral vestibular dysfunction. The average asymmetry of bilateral vestibular in the UVH group was 47.06% (ranging from 24.59% to 89.01%). None of the patients suffered from severe vertigo (which might impact the gait pattern) during the week before the test. Among these subjects, 7 patients reported a falling tendency during locomotion. The UVH patients showed clear cognitive status, normal visual status, sound cardiopulmonary function, and no rheumatoid arthritis or osteoporosis according to their past medical history. The normal subjects showed clear cognitive status, normal visual status, and no history of fall. The results of vestibular function examination in the control group were negative. Participant information is shown in Table 1.

### 2.2. Experimental Setup

Vestibular function was evaluated by the Fitzgerald and Hallpike bithermal caloric test [27]. A video-based system (Ulmer VNG, Version 1.4, Synapsys, Marseille, France) was used to record the nystagmus after each irrigation of 30° or 44° air to the unilateral side of the external auditory canal. The maximum slow speed phase of nystagmus was used to calculate the unilateral vestibular canal paresis (CP) using Jongkees' formula [28].

**Figure 1:**
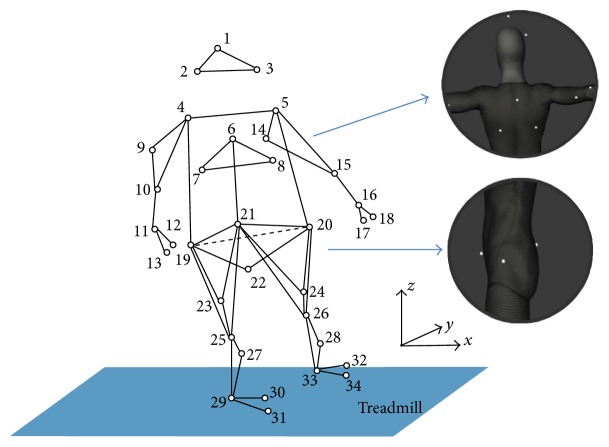
Location of the 34 anatomic markers and defined direction of movements used in this study. The trunk segment was represented by marker 6 (first thoracic vertebrae, T1), marker 7, and marker 8. The hip segment was represented by marker 19, marker 20 (anterior superior iliac spine, ASIS), and marker 22. The positive *x*-axis is the direction of walking, the positive *y*-axis is the left direction, and the positive *z*-axis is upward.

In the experiments, a total of thirty-four retroreflective 9.5 mm markers were attached to the anatomic positions of the subjects (Figure 1). The 3D coordinates of the marker trajectories were recorded using a six-camera optical motion capture system sampling at 120 Hz (OptiTrack, NaturalPoint Inc., OR, USA, Flex 13). Subjects were instructed to walk on a treadmill (2 m × 0.5 m, BH Fitness, NSW, Australia) for at least 50 strides. Each subject was tested at their preferred walking speed (PWS) to eliminate the influence of speed on gait stability [29]. The subjects were required to look forward and stare at the white screen during treadmill walking. Sufficient but not dazzling light environment should be ensured to minimize the interindividual difference caused by visual information. The subjects wore standard laboratory shoes during the test. The participants were given sufficient time to become comfortable with walking on the treadmill before gait pattern data was collected. During the procedure, the experimenter stopped the treadmill if the subject encountered any sort of difficulty.

### 2.3. Data Processing

The raw data of coordinates was low-pass-filtered with a cut-off frequency of 2 Hz by a zero-lag Butterworth filter [20]. Kinematic parameters (e.g., stride width and cadence) were defined by the position of the markers located on the heel and big toe. A complete stride was defined as the time between heel contact with the surface and the subsequent contact on the same side. The range of motion (ROM) was calculated from the difference between the maximum and minimum joint angles. The standard deviation (SD) between gait cycles was calculated for the trunk and hip kinematics to reflect the dispersion degree of motion.

Nonlinear time series analysis was applied to quantify the local dynamic stability of the gait pattern based on state-space representation of each time series [29, 30]. The nonlinear time series of trajectories was collected when the participants were walking continually on the treadmill. The marker attached to T1 was chosen to represent the whole body, and the trajectories of the T1 marker in anterior-posterior (AP), medial-lateral (ML), and vertical (VT) directions were taken as three one-dimensional random time series. According to Takens's theory [31], each one-dimensional temporal-spatial series was reconstructed to higher dimensional state space *S*(*t*) using time-delayed copies. A valid state space is any vector space containing numerous independent coordinates.

(1) $$\begin{array}{c} S\left(t\right)={\left[x\left(t\right),x\left(t+\tau \right),\ldots ,x\left(t+\left(n-1\right)\tau \right)\right]}^{T}, \end{array}$$

where *x*(*t*), the one-dimensional random time series, was time-normalized using a shape-preserving spline interpolation. The 50 contiguous strides were resampled to 10,000 data points by interpolation, and *τ* was time delay chosen as 50 sample copies [32, 33]. *n* represents the embedding dimension, which was set as 5 according to a previous study that had applied a global false nearest neighbor (GFNN) analysis [20] to determine the minimum number of embedding dimensions to fully unfold the one-dimensional data. In the state space, the Euclidean distances between neighboring trajectories (di(*t*)) diverge at an exponential rate, which is quantified by the Lyapunov exponent (*λ*_1_), and the definition of (*λ*_1_) is shown as (2)

(2) $$\begin{array}{c} di\left(t\right)=De^{\lambda_{1}t}, \end{array}$$

where *D* is the initial separation displacement between trajectories and di(*t*) represents the mean displacement between neighboring trajectories in the reconstructed state space at time *t*. According to a previously published algorithm [34], for each point *i* in the state space, di(*t*) was measured for each pair of the nearest neighbors over 10 subsequent strides. When *t* → ∞ and *D* → 0 at the same time, taking the natural log of (2) results in (3):

(3) $$\begin{array}{c} In\left[d_{j}\left(i\right)\right]=\lambda_{1}△t. \end{array}$$

*λ*_s_ is estimated from the slopes of linear fits for a period of 0 to 1 stride (△*t*); the higher *λ*_s_ implies the poorer local dynamic stability of the gait pattern.

All the data values were presented as mean ± SD. Significant differences in joint angle, rotation angle, *λ*_s_ between the UVH and control groups were determined by independent samples *t*-test. Also, linear regression analysis was performed to test the relationship between the gait parameters and CP value. Differences were considered statistically significant if *P* < 0.05. The data was calculated from coordinates of markers by MATLAB (version 2013a, The MathWorks BV, Natick, USA). All statistical analyses were performed using SPSS (version 22.0, SPSS Inc., Chicago, USA).

## 3. Results

Table 2 shows the differences between the UVH and control group in PWS, stride width, cadence, and angular range of joint motion. Significantly wider stride width was observed in the UVH group than the control group (*P* < 0.05). The ankle motion exhibited significantly less flexibility in the UVH group than in the control group (*P* < 0.05). No other differences were found between the two groups.

**Figure 2:**
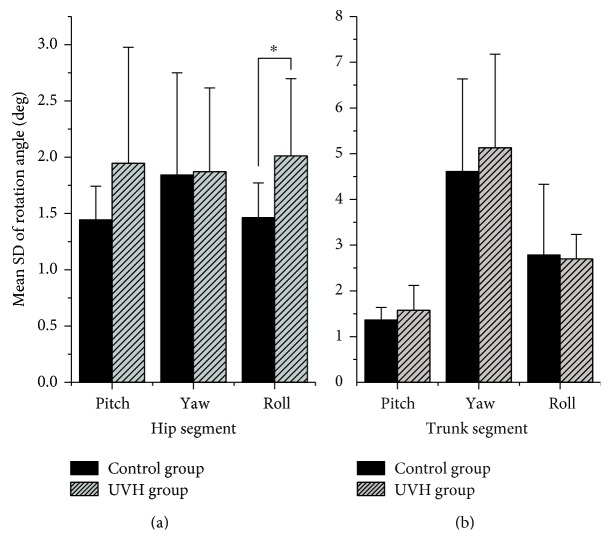
Mean SD of rotation angles in the hip (a) and trunk (b) segments. Asterisks indicate a significant difference (*P* < 0.05) between the two groups.

**Figure 3:**
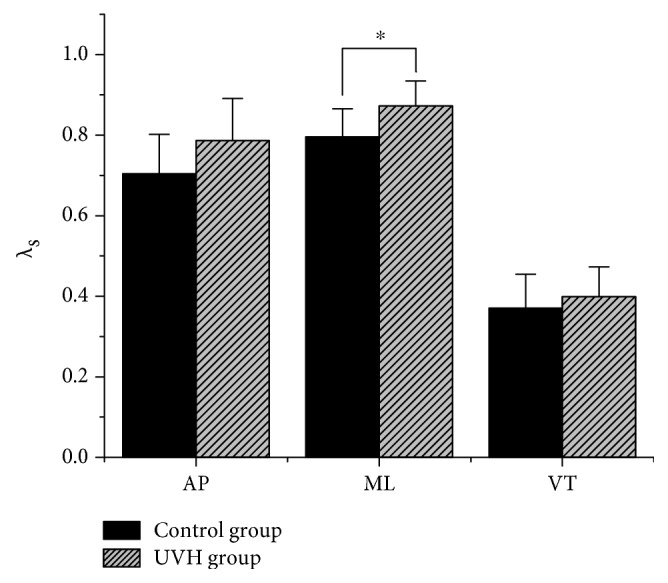
Mean *λ*_s_ values in all three directions. Asterisks indicate a significant difference (*P* < 0.05) between the two groups. Error bars show the standard deviation of *λ*_s_.

Higher gait variability was observed from the mean SD of rotation angle in the UVH group than the control group, especially on the coronal (side-to-side) plane of hip motion. The roll rotation variability was significantly higher in the UVH group than the control group (*P* < 0.05). No other significant differences in rotation angle were detected (Figure 2). The *λ*_s_ of the UVH group was significantly higher than that of the control group in the ML direction (*P* < 0.05). The *λ*_s_ in the AP and VT directions was not significantly different between the groups (Figure 3).

**Figure 4:**
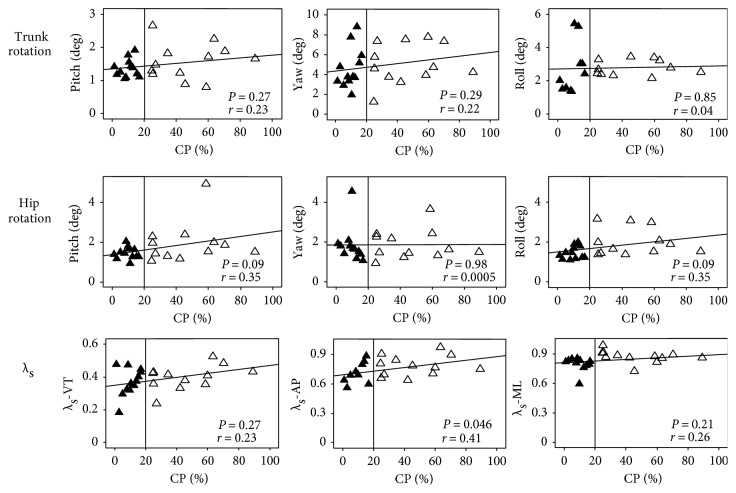
Linear regression analyses of gait parameters (mean SD and *λ*_s_) and canal paresis (CP) value. The black triangles represent control group participants, whereas the white triangles represent UVH group participants. The perpendicular reference line represents the CP threshold value (20%), above which subjects are considered having unilateral vestibular dysfunction. The linear relationship between gait parameters and CP value was significant when *P* < 0.05.

Linear regression analysis was used to estimate the relationship between motor output parameters (mean SD of rotation angles and *λ*_s_) and CP. We identified significant linear trends between *λ*_s_ and CP in the AP direction (*P* < 0.05, *R* = 0.41) but not in the ML or VT directions. No other significant linear relationships were found between mean SD of rotation angles and CP (Figure 4).

## 4. Discussion

The vestibular apparatus can detect motion of the head and generate a sensory input to the sensorimotor control, which plays an important role in adjusting stride-to-stride limb trajectories, thereby maintaining balance and smoothing unintended irregularities during walking [19]. Here, kinematic variability and the local dynamic stability of walking were used to quantify the impact of vestibular impairment on the gait pattern. We found that UVH participants expanded their stride width and reduced ankle joint motion range to cope with the feelings of instability or dizziness. Similarly, in visually and mechanically destabilizing environments, normal subjects exhibited shorter and wider steps as a result of the interferential visual and proprioceptive input [35]. Therefore, this kind of “cautious gait mode” may be a coping strategy shared across various sensory input errors. Elderly adults that adopt a “cautious gait mode” have higher acceleration variability and fall risk [11, 21, 25]. Here, the stride-to-stride variability of the roll rotation of the hip joint was also significantly higher in the UVH group than the control group (Figure 2()). The hip, as a connection joint between the upper body and the lower limb, supports the mass of the body and thus plays an important role in gait performance. The vestibular system contributes important information to regulate the motion in the ML direction, and the unilateral vestibular hypofunction may be predominantly responsible for impaired stability in the coronal plane [36, 37]. Thus, the roll rotation in the hip joint was sensitive to the bilateral vestibular inputs and inevitably affected the motion of the lower limb [7], which was consistent with the finding in our study. Higher variability was associated with more unstable gait in several studies, which indicates increasing fall risk of gait in several studies [18, 38, 39]. However, lower variability has also been associated with the unstable gait with the impaired mobility, for example, in cases where individuals are unable to make adequate adjustments in response to changes in the environment. Maki reported decreased step width variability in fall-prone subjects [39]. Similarly, Bruijn et al. reported a lower variability of trunk accelerations in the ML direction in frail elderly subjects compared to healthy elderly subjects [24]. Furthermore, Dingwell and Marin found the greater variability of kinematics at both faster and slower walking speeds [29], whereas Kang and Dingwell proposed that fall risk would increase with walking speed [22]. Taken together, the above-mentioned studies implied that variability of kinematics might not directly associate with fall risk.

Local dynamic stability has been used to assess fall risk associated with unstable gait and reported as having excellent validity [19, 29]. The local dynamic stability adopted here quantifies how the neuromuscular system responds to local perturbations (of either internal [e.g., neuromuscular] or external [e.g., the wind, surface friction, or uneven surfaces] sources) during gait [24]. During the long walking period, the loss of gait stability is a gradual process that accumulates small perturbations until eventually overcoming a normal gait mode. Thus, local dynamic stability (i.e., *λ*_s_) can be used to characterize fall risk. In the ML direction, patients in this study showed significantly poorer local dynamic stability of walking than control participants (Figure 3()); the patients with UVH were more sensitive to the perturbations due to sensory hypofunction. After the vestibular injury, vestibular cues cannot be normally integrated with visual and somatosensory cues, producing errors in the processing of sensory inputs in the central nervous system [6, 40]. Local dynamic stability was different between the two studied groups in the ML direction, but not in the AP or VT directions. This finding is in line with the previous studies that lateral gait stability was associated with asymmetry of bilateral sensory inputs [7, 21]. However, Dingwell et al. [19] and Dingwell and Cavanagh [41] found the opposite results in patients with diabetic neuropathy. The slow progression of diabetic neuropathy provides a long period for patients to develop compensation mechanisms for a range of local perturbations, whereas the acute nature of vestibular hypofunction can lead to sudden neuromuscular control errors, with which the patients are unable to cope. Similarly, the local dynamic stability of normal subjects decreases when confronted with sudden environmental or sensory perturbations [7, 8, 20]. Therefore, it seems reasonable that acute UVH patients exhibit poorer local dynamic stability and thus have higher fall risk.

We identified a significant correlation between CP and *λ*_s_ in the AP direction but no such correlation in the ML direction (despite patients having significantly poorer local dynamic stability in the ML direction). This may be due to the limited number of participants in this study. The variability of rotation angle and *λ*_s_ showed the increasing trends with increasing vestibular functional asymmetry which reflect they can be applied clinically in the characterization of gait disorders for UVH patients. The limitation of this study is that gait on treadmill is not equal to natural gait over ground. The influence of vestibular impairment on gait patterns may not entirely exhibit during treadmill walking. Besides that other sensory inputs may still affect gait patterns, visual or somatosensory information could conceal the effect of vestibular functional disorders. The overground gait analysis of patients with visual deprivation should be designed in the future to explore the vestibular contribution without the effect of other sensory information.

## 5. Conclusion

Here, we verified the linear relationship between vestibular functional asymmetry and local dynamic stability. Variability and local dynamic stability might be useful for assessing the degree of vestibular functional asymmetry, and more subjects should be involved in further studies to investigate the relationship between vestibular functional asymmetry and gait disorders.

## Figures and Tables

**Table 1:** Participant information.

Number	Sex	Age	Diagnosis	Vestibular functional asymmetry
				Canal paresis (%)
(1)	F	59	MD	34.47
(2)	F	66	TA	63.35
(3)	M	52	MD	25.1
(4)	F	60	MD	26.95
(5)	M	61	SD	42.2
(6)	M	62	SD	70.18
(7)	M	69	MD	45.32
(8)	F	68	MD	89.01
(9)	M	65	MD	24.59
(10)	M	53	MD	58.43
(11)	M	52	MD	59.84
(12)	M	29	MD	25.23

The description of information and clinical vestibular measures in the UVH group. MD: Meniere's disease; TA: tinnitus aurum; SD: sudden deafness.

**Table 2:** Kinematic characteristics (mean ± SD) of the UVH and control groups. Asterisks indicate a significant difference (*P* < 0.05) between the two groups. Stride width was normalized to the hip width of each subject.

	UVH group	Control group	*P* value
PWS (km/h)	1.91 ± 0.35	2.22 ± 0.83	0.12
Normalized stride width (%)	0.79 ± 0.05	0.74 ± 0.06	0.03^∗^
Cadence (steps/min)	0.79 ± 0.07	0.84 ± 0.10	0.18
Range of knee motion (deg)	50.83 ± 6.85	52.27 ± 7.33	0.63
Range of ankle motion (deg)	17.57 ± 2.54	21.47 ± 5.64	0.04^∗^
